# Altering Knee Abduction Angular Impulse Using Wedged Insoles for Treatment of Patellofemoral Pain in Runners: A Six-Week Randomized Controlled Trial

**DOI:** 10.1371/journal.pone.0134461

**Published:** 2015-07-31

**Authors:** Ryan T. Lewinson, J. Preston Wiley, R. Neil Humble, Jay T. Worobets, Darren J. Stefanyshyn

**Affiliations:** 1 Human Performance Laboratory, Faculty of Kinesiology, University of Calgary, Calgary, Alberta, Canada; 2 Biomedical Engineering Program, Schulich School of Engineering, University of Calgary, Calgary, Alberta, Canada; 3 Cumming School of Medicine, University of Calgary, Calgary, Alberta, Canada; 4 Sport Medicine Centre, Faculty of Kinesiology, University of Calgary, Calgary, Alberta, Canada; Emory University School Of Medicine, UNITED STATES

## Abstract

**Objective:**

Determine if a change in internal knee abduction angular impulse (KAAI) is related to pain reduction for runners with patellofemoral pain (PFP) by comparing lateral and medial wedge insole interventions, and increased KAAI and decreased KAAI groups.

**Design:**

Randomized controlled clinical trial (ClinicalTrials.gov ID# NCT01332110).

**Setting:**

Biomechanics laboratory and community.

**Patients:**

Thirty-six runners with physician-diagnosed PFP enrolled in the trial, and 27 were analyzed.

**Interventions:**

Runners with PFP were randomly assigned to either an experimental 3 mm lateral wedge or control 6 mm medial wedge group. Participants completed a biomechanical gait analysis to quantify KAAIs with their assigned insole, and then used their assigned insole for six-weeks during their regular runs. Usual pain during running was measured at baseline and at six-week follow-up using a visual analog scale. Statistical tests were performed to identify differences between wedge types, differences between biomechanical response types (i.e. increase or decrease KAAI), as well as predictors of pain reduction.

**Main Outcome Measures:**

Percent change in KAAI relative to neutral, and % change in pain over six weeks.

**Results:**

Clinically meaningful reductions in pain (>33%) were measured for both footwear groups; however, no significant differences between footwear groups were found (*p* = 0.697). When participants were regrouped based on KAAI change (i.e., increase or decrease), again, no significant differences in pain reduction were noted (*p* = 0.146). Interestingly, when evaluating absolute change in KAAI, a significant relationship between absolute % change in KAAI and % pain reduction was observed (*R* ^2^ = 0.21; *p* = 0.030), after adjusting for baseline pain levels.

**Conclusion:**

The greater the absolute % change in KAAI during running, the greater the % reduction in pain over six weeks, regardless of wedge type, and whether KAAIs increased or decreased. Lateral and medial wedge insoles were similar in effectiveness for treatment of PFP.

**Clinical Relevance:**

Altering KAAI should be a focus of future PFP research. Lateral wedges should be studied further as an alternative therapy to medial wedges for management of PFP.

**Trial Registration:**

ClinicalTrials.gov NCT01332110

## Introduction

Patellofemoral pain (PFP) is the most common running injury, affecting approximately 13% of all runners [1–3]. It is characterized by peri and/or retro patellar pain, and can be so debilitating that everyday tasks become difficult [4,5]. Additionally, it is a chronic condition, with 50% of patients reporting same or worse pain after 4 years, and 27% still reporting same or worse pain after 16 years [6]. Moreover, it has been proposed that when left untreated, PFP may lead to the development of patellofemoral osteoarthritis [7,8]. Patellofemoral pain is commonly treated with medially wedged insoles, but the reason why this type of footwear produces clinical benefits is unknown. Indeed, systematic reviews have highlighted the need for randomized controlled trials studying the relationship between wedged insoles, biomechanics and treatment outcomes [9,10].

It was previously found that subjects with PFP demonstrate increased internal knee abduction angular impulses (KAAIs) compared to healthy controls during running [11]. Mathematically, KAAIs are calculated as the integral of the internal knee abduction moments with respect to stance time. Therefore, biomechanically, KAAIs represent the cumulative resultant frontal-plane internal load the knee joint experiences during the stance phase of running. Internal knee abduction moments develop to balance external moments acting to compress the medial tibiofemoral joint. To counteract the external moment, it is believed that tension increases, passively or actively, in lateral knee tissues such as muscles/tendons or retinacula, and this may create greater compressive and shear stress in the patellofemoral joint, activating mechanical nociceptors within the joint thereby contributing to PFP [4,11]. Thus, one possible strategy for PFP treatment is a reduction of KAAIs during running.

Laterally wedged insoles tend to decrease KAAIs during locomotion in healthy subjects via a lateral shift of the centre of pressure beneath the foot [12–14]. In the osteoarthritis literature, lateral wedges have also been shown to reduce external knee adduction moments (a variable mathematically related to internal knee abduction moments and angular impulses) [15, 16]. Despite these positive biomechanical effects, lateral wedges have never been tested as a treatment for PFP. Conversely, medial wedges are commonly used for treatment of PFP [9, 17, 18]; however these types of wedges have been shown to increase internal and external frontal-plane knee joint moments during locomotion in both healthy and knee osteoarthritis populations [14, 19, 20, 21]. Thus, medial wedges tend to increase frontal plane knee loading–a characteristic associated with PFP–yet still seem to be of benefit to patients with PFP, although KAAI and clinical outcomes have never been evaluated together previously in insole studies for individuals with PFP. It is therefore not currently clear whether a reduction or increase in KAAI magnitude is more beneficial for pain relief.

We undertook a randomized controlled trial to determine if a change in KAAI is related to pain reduction for runners with PFP, specifically by comparing lateral and medial wedge insole interventions, and increased KAAI and decreased KAAI groups. It was hypothesized that lateral wedges would be a superior intervention to medial wedges as a result of reduced KAAI magnitudes. In the event that not all individuals experienced KAAI changes in the expected directions (i.e. reduction for lateral wedge and increase for medial wedge), we hypothesized that those who did experience reduced KAAIs would experience greater pain reductions than those who experienced increased KAAIs. Among all participants, we hypothesized a linear relationship between % change in KAAI and % change in pain over the six-week period, with greater % reductions in pain attributed to those runners who experience greater % reductions in KAAI.

## Materials & Methods

### Participants

**Fig 1:**
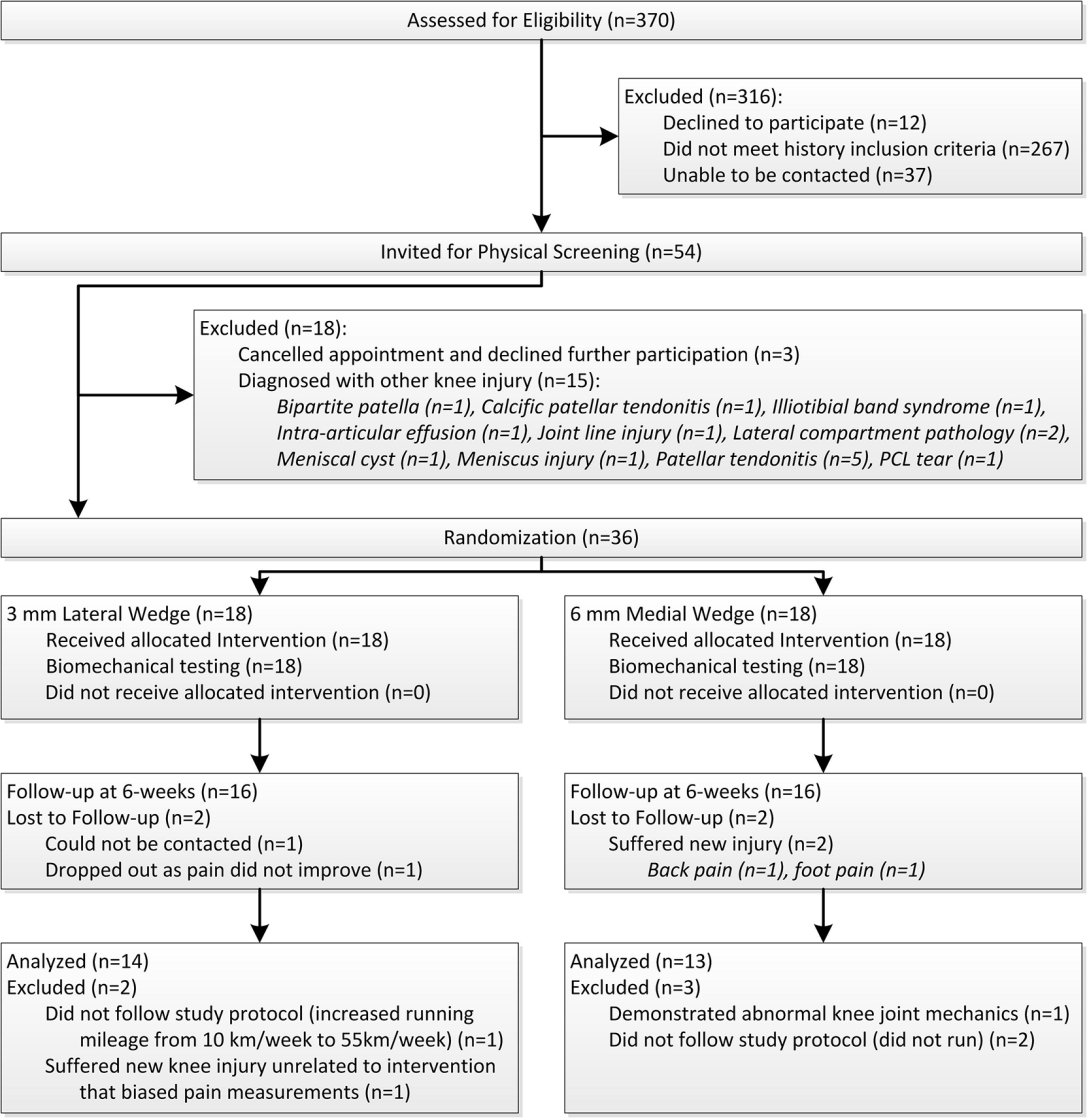
CONSORT diagram showing subject flow through trial.

Volunteers responded to television, newspaper, radio and poster advertisements calling for runners who suffered from knee pain. Volunteers were first screened by telephone or email correspondence according to the PFP history criteria outlined in Table 1 [11,22]. Upon satisfying these history criteria, volunteers were invited for assessment by a sports medicine physician, who diagnosed PFP and confirmed eligibility for participation using the abovementioned history criteria, as well as additional physical criteria (Table 1) [11,22]. A diagram showing participant flow through the trial can be found in Fig 1.

**Table 1:** History and Physical Inclusion/Exclusion Criteria. The criteria used to diagnose patellofemoral pain syndrome and participant eligibility in this study are shown.

Patellofemoral Pain Syndrome Diagnostic Criteria
**History Criteria**
1. Nontraumatic unilateral and/or bilateral peripatellar or retropatellar knee pain.
2. Patellofemoral knee pain with and/or after activity for at least one month.
3. Inactivity patellofemoral pain and/or stiffness, especially with sitting with knees held in flexed posture (not mandatory; patients must demonstrate at least #2).
4. No other health conditions that could influence study results or compromise patient safety (ex. arthritis, recent foot/ankle/knee/hip injury, cardiovascular disease etc.).
5. Have not undergone any form of knee surgery or arthroscopy within the last two years.
6. Run at least 15km per week for the past 4 weeks.
**7**. Be between 18 and 50 years old.
Not currently using any form of treatment for pain (within last 3 weeks).
**Physical Criteria**
1. No or minimal articular or periarticular effusion or bursitis.
2. No significant joint line tenderness.
3. No knee ligamentous instability.
4. Peripatellar tenderness ± mild inferior patellar pole tenderness.
5. No patellar apprehension.
6. No limitation of hip movement suggesting osteoarthritis.
7. No limitation of talocrural or subtalar joint motion.

### Interventions

**Fig 2:**
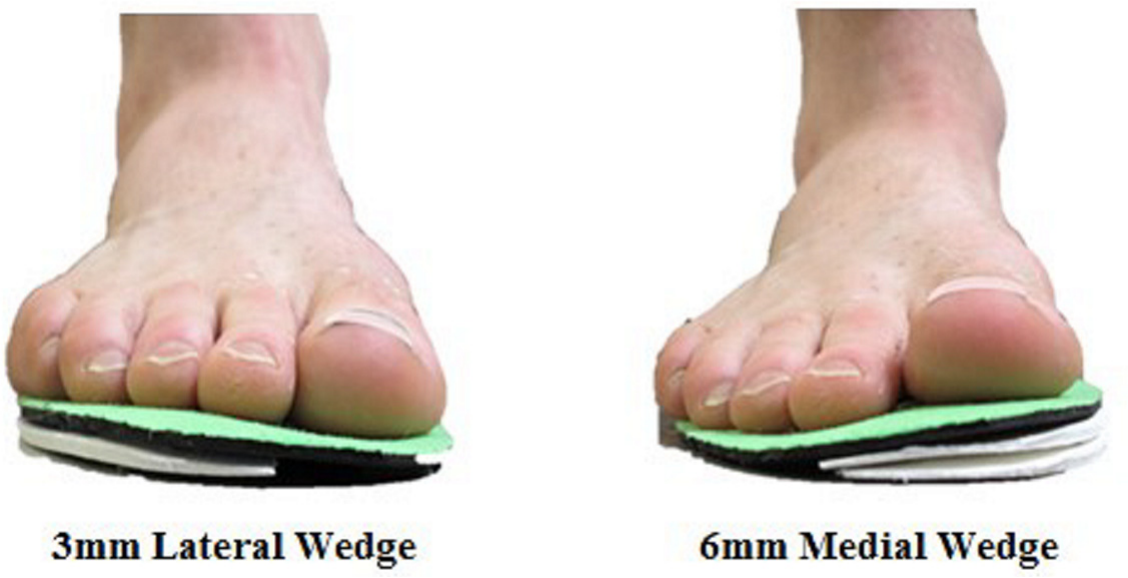
The wedged insole conditions tested in this study. On right, the 6mm medial wedge is shown. On left, the 3mm lateral wedge is shown.

All wedged insoles were made by a podiatrist (>10 years experience). Insoles were prepared to the same specifications a priori. Insoles were cut to the appropriate shoe size out of 3 mm ethylene vinyl acetate “EVA” (SOLFLEX Firm 60, Vittoria Phoenix Inc., Vittoria, Ontario), and then sanded either medially or laterally along the full length of the insole to create a wedge. Thicker wedges were made by placing additional EVA layers on top of one another. We compared a 3 mm lateral wedge to a 6 mm medial wedge, which corresponded to wedge angles of approximately 3° and 6° respectively (Fig 2). A medial wedge of 6 mm was chosen since this is commonly studied in PFP research, is often used clinically and since greater thicknesses may be uncomfortable [17,18]. Additionally, other studies using them have shown good subject compliance [17]. Thus the 6 mm medial wedge represented the gold-standard wedge for our study. A 3 mm lateral wedge was chosen as a conservative thickness to ensure a comfortable fit within the shoe, and to reduce the risk of any injurious effects since lateral wedges have not been tested as a treatment for PFP previously. Additionally, wedge thicknesses lower than 3 mm may not elicit KAAI changes during gait [23]. Since measures of static joint alignment, foot shape, or patellar tracking were not performed in this study, these factors were not considered in wedge design or wedge assignment to participants.

Insoles were placed under the sock-liner of a commercially available light-weight neutral running shoe without any additional foot support features (adiZero Mana, adidas International, Herzogenaurach, Germany). By providing each subject with the same shoe, and altering only the non-custom insole that was placed within, biomechanical and pain measurement errors that could result due to different running shoe models, or different custom-fit orthoses shapes were eliminated. Participants were fitted to male size 8–12 or female size 6–10 shoes.

### Randomization & Masking

A block-randomization sequence was generated prior to commencing the study using a random number generator to allocate insole conditions in a 1:1 ratio. A block size of six was used, and a separate block was used for males and females to ensure even sex distributions. Subjects received their allocated insole following baseline data collection by a single researcher. Since the randomization sequence was generated a priori, the researcher was not blinded during data collection. Although subjects could feel and see their assigned intervention during the six-week study, all subjects were told they were receiving an experimental insole, regardless of the condition they were actually assigned to. Physicians responsible for PFP diagnosis were blinded as to which group the participant would be assigned. All researchers were blinded during data analysis.

### Protocol

Baseline characteristics were recorded in a biomechanics laboratory. Subjects indicated their usual pain during running on a computerized visual analog scale (cVAS) with the terms “no pain” and “worst pain possible” used as anchors at the 0 and 100 positions, respectively [24].

**Fig 3:**
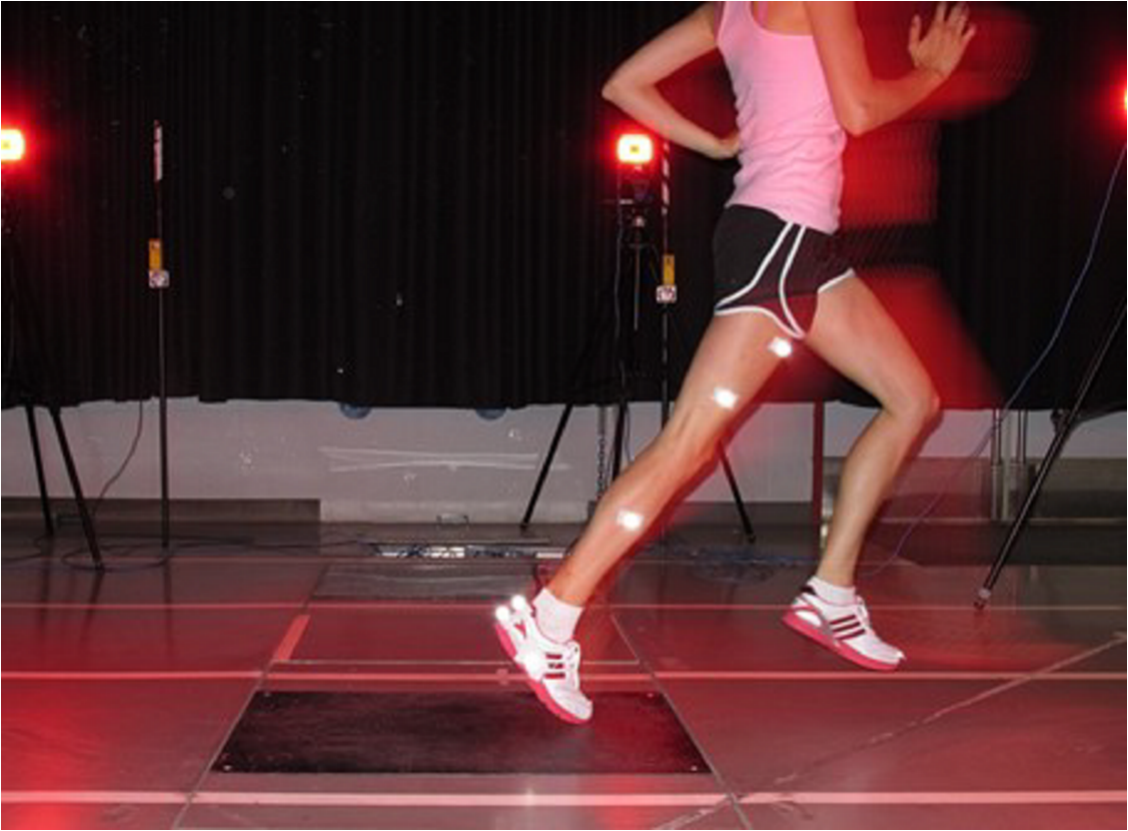
Photograph of an experimental trial occurring. The motion capture cameras, force platform, timing system, and retroreflective markers can be seen.

Three retroreflective markers were then secured to each of the shoe, shank and thigh of each subject’s most symptomatic limb using two-sided tape. As subjects ran along a 30 m runway, eight Motion Analysis cameras (Motion Analysis Corp., Santa Rosa, California) recorded the 3D trajectories of the retroreflective markers at a frequency of 240 Hz, while a force platform (Kistler AG, Winterthur, Switzerland), mounted flush with the lab floor, simultaneously recorded ground reaction force data at a frequency of 2400 Hz (Fig 3). Two photocells were used to ensure running speed was within the target range of 4.0±0.2 m/s [11,14]. In a randomly assigned order, subjects completed five trials wearing their assigned intervention footwear and five trials wearing the neutral running shoe with wedged insoles removed. Subjects were provided with breaks between trials and time to warm-up with each shoe before testing. Standing neutral trials, where additional markers were placed over the lateral and medial epicondyles and malleoli, were also recorded with each footwear condition to define joint centres, and segment lengths. These data were used to estimate segment centres-of-mass and segment moments of inertia [25–27]. The point midway between each set of additional markers defined the knee and ankle joint centres, respectively [28].

Following biomechanical testing, we provided subjects with their assigned footwear (neutral shoe and assigned insole), and asked them to use this footwear during their regular runs for six-weeks. Subjects were asked not to alter their running frequency or mileage during the trial [29], and asked to avoid co-interventions. Each week, subjects completed an online survey from their own home to document running mileage, running frequency, co-interventions, adherence and side effects. On the sixth week, subjects completed an exit cVAS survey from home to determine the effects of the insole intervention on usual pain during running. Only running pain was assessed in this study since participants only wore the footwear during their runs, and since the biomechanical variable of interest (KAAI) was collected during running.

The study was designed according to CONSORT guidelines (S1 CONSORT Checklist). The protocol (S1 Protocol) was approved by the University of Calgary’s Conjoint Health Research Ethics Board prior to recruitment and all subjects gave informed written consent prior to participation. The trial was registered with ClinicalTrials.gov (ClinicalTrials.gov ID# NCT01332110).

### Data Processing

Pain and biomechanical data were processed once the trial completed. Baseline and six-week cVAS scores, and weekly survey responses were downloaded from a secure server. Kinetic and kinematic biomechanical data were imported into KinTrak v7.0 software (University of Calgary, Calgary, Alberta), and were smoothed using fourth-order Butterworth low-pass filters, with cut-off frequencies of 50 Hz and 12 Hz, respectively [30,33]. Three-dimensional joint kinematics were determined using a joint coordinate system, and resultant 3D joint kinetics were calculated using inverse dynamics [25,28,34,35]. Resultant internal knee abduction moments were integrated with respect to stance time to yield the knee abduction angular impulse (KAAI) for each trial. The average KAAI across the five trials in each insole condition for each subject was used in subsequent statistical analysis. Errors associated with calculation of KAAIs are described elsewhere [25,28].

### Outcome Measures

The primary outcome measures for this study were (1) change (i.e. difference) in KAAI between the neutral shoe condition and the assigned wedge condition, measured by biomechanical gait analysis, and (2) change in pain during running over the six-weeks measured by cVAS. Change scores were quantified as % change, since this method is less biased by baseline scores [36].

### Sample Size

Collins et al.[17] reported that participants in their medial wedge condition experienced usual pain of 38.6 mm (SD 16) at baseline and 25.4 mm (SD 17.4) at six-week follow-up on 100 mm VASs, or approximately a 34% decrease in pain. We considered the lateral wedge condition to be a superior treatment for PFP if pain could be reduced by a clinically meaningful additional 20 points (i.e. a 86% reduction in pain) [36,37]. Based on this mean difference, a standard deviation of 17.4, a two-tailed α of 0.05, and an expected drop-out rate of 15%, 13 subjects per group would achieve 80% power in comparing the lateral and medial wedge groups by independent-samples t-test.

### Statistics

All statistical analyses were conducted using SPSS v19 (IBM Corp., Somers, NY) on a per-protocol basis. All data were first checked for normality. The primary analysis for this study was a multiple linear regression to compute the r-squared value (*R* ^*2*^), where % change in KAAI, and baseline pain were predictor variables, and % change in pain during running was the outcome variable. Since medial wedges are known to increase frontal-plane knee loads, yet may still be beneficial for PFP treatment [17, 18], we also conducted a second multiple regression analysis where absolute % KAAI (instead of ± % change) and baseline pain were predictor variables, and % change in pain was the outcome variable. This second analysis allowed us to observe relationships between any type of change in KAAI, regardless of increase or decrease.

Two-tailed independent-samples t-tests were used to compare all baseline and follow-up data between insole groups. In addition to analyzing participants grouped by assigned insole, comparisons were also made between an “increased KAAI” group that comprised all participants who experienced increased KAAIs, regardless of wedge assigned, and a “decreased KAAI” group that comprised all participants who experienced decreased KAAIs, regardless of wedge assigned. This secondary grouping allowed us to make the distinction between differences that occurred due to wedge type and differences that occurred due to wedge biomechanical effect. A significance level of 0.05 was used for all statistical tests.

## Results

From June to October 2011, 370 volunteers were screened, of which 36 were enrolled in the study (Fig 1). Reasons for participant exclusion are outlined in Fig 1. The trial completed in December 2011, with 27 subjects deemed eligible for subsequent analysis. Baseline characteristics for both wedge groups and both KAAI groups were comparable (Table 2). Baseline pain was not significantly different between medial and lateral wedge conditions (*p* = 0.211) or between increased and decreased KAAI groups (*p* = 0.862). Across all 27 runners who completed the trial, weekly running distance remained unchanged over the 6-week period (18.3±9.2 km at baseline vs. 20.2±8.9km at 6-weeks, *p* = 0.359) as did weekly running frequency (3.7±1.8 days/week at baseline vs. 4.0±1.7 days/week at 6-weeks, *p* = 0.327)

**Table 2:** Baseline characteristics. Baseline characteristics are shown for each footwear group, and the regroupings*. Baseline measures were compared using paired-samples two-tailed t-tests (α = 0.05).

	Initial Randomization			Regrouping*		
Variable	3 mm Lateral Wedge (n = 14)	6 mm Medial Wedge (n = 13)	*p*-value	Decreased KAAI (n = 13)	Increased KAAI (n = 14)	*p*-value
Physical Characteristics:						
*Mean (SD) Age [years]*	33.6 (9.9)	28.6 (8.7)	0.181	33.2 (9.1)	29.3 (9.8)	0.290
*Mean (SD) Body mass index [kg/m* ^*2*^ *]*	24.5 (3.3)	23.6 (4.3)	0.553	25.1 (4.4)	23.0 (2.9)	0.150
*Mean (SD) Height [cm]*	172.1 (9.6)	174.5 (5.2)	0.442	174.8 (6.2)	171.9 (8.9)	0.344
*Mean (SD) Mass [kg]*	73.2 (15.4)	71.8 (13.2)	0.805	76.7 (14.0)	68.6 (13.6)	0.137
*# male/female*	5/9	6/7	-	6/7	5/9	-
*# unilateral/bilateral pain*	9/5	4/9	-	7/6	6/8	-
**Running History Characteristics:**						
*Mean (SD) weekly running days [days]*	4.14 (1.7)	3.15 (1.8)	0.157	4.0 (2.0)	3.36 (1.6)	0.363
*Mean (SD) weekly running distance [km]*	21.3 (9.9)	15.1 (7.5)	0.080	20.7 (10.5)	16.1 (7.5)	0.201
*Mean (SD) running experience [years]*	6.75 (6.3)	8.54 (8.7)	0.545	5.12 (4.3)	9.93 (9.1)	0.095
**Baseline Biomechanics with Neutral Shoe:**						
*Mean (SD) KAAI [Nms]*	14.2 (6.5)	17.3 (5.1)	0.187	18.0 (5.4)	13.6 (5.9)	0.053
**Pain by cVAS at Baseline:**						
*Mean (SD) pain*: *Running [0–100]*	52.5 (19.4)	41.5 (22.4)	0.183	44.0 (18.2)	50.1 (24.1)	0.464

* subjects were regrouped based on whether they experienced increased or decreased KAAIs with their assigned footwear, regardless of wedge type

### Outcome Measures

A clinically meaningful reduction in pain of 33% [29], or greater, was found for both the lateral and medial wedge groups, and both the increased and decreased KAAI groups (all participants except for two experienced a reduction in pain). Interestingly, when comparing between wedge conditions, or between KAAI change conditions, no differences were observed in terms of the % change in pain from baseline (Table 3).

**Table 3:** Main results of this study are displayed for each footwear group, and the regroupings* after the six-week intervention. Mean differences between groups are shown with 95% confidence intervals. Negative % change values indicate that there was a decrease from baseline.

**Initial Randomization**				
**Variable**	**3 mm Lateral Wedge (n = 14)**	**6 mm Medial Wedge (n = 13)**	**Mean Difference (95% C.I.)**	***p*-value**
**Intervention Biomechanics:**				
*Mean (SD) KAAI [Nms]*	14.0 (6.2)	17.45 (5.5)	-3.45 (-7.87 to 0.97)	0.183
*Mean (SD) KAAI [% change from week 0]*	‒0.27 (8.6)	1.34 (17.3)	-1.61 (-11.8 to 8.59)	0.759
*# decreased/increased KAAI*	6/8	7/6	N/A	N/A
*# lateral/medial wedge*	N/A	N/A	N/A	N/A
**Pain by cVAS at 6-weeks:**				
*Mean (SD) pain*: *Running [0–100]*	23.3 (17.7)	9.5 (10.7)	13.8 (2.60 to 24.9)	†0.023
*Mean (SD) pain*: *Running [% change from week 0]*	-55.8 (32.3)	-62.1 (49.4)	6.3 (-25.0 to 37.6)	0.697
*# improved/worsened/no change*	13/1/0	12/1/0	N/A	N/A
**Regrouping** *				
**Variable**	**Decreased KAAI (n = 13)**	**Increased KAAI (n = 14)**	**Mean Difference (95% C.I.)**	***p*-value**
**Intervention Biomechanics:**				
*Mean (SD) KAAI [Nms]*	16.2 (5.1)	15.1 (6.9)	1.1 (-3.47 to 5.72)	0.637
*Mean (SD) KAAI [% change from week 0]*	‒10.0 (5.5)	10.3 (10.6)	-20.3 (-13.9 to -27.0)	†<0.001
*# decreased/increased KAAI*	N/A	N/A	N/A	N/A
*# lateral/medial wedge*	6/7	8/6	N/A	N/A
**Pain by cVAS at 6-weeks:**				
*Mean (SD) pain*: *Running [0–100]*	12.7 (13.0)	20.4 (18.2)	-7.7 (-19.7 to 4.35)	0.222
*Mean (SD) pain*: *Running [% change from week 0]*	-70.7 (24.7)	-47.7 (49.8)	-23.0 (-53.0 to 7.0)	0.146
*# improved/worsened/no change*	13/0/0	12/2/0	N/A	N/A

* subjects were regrouped based on whether they experienced increased or decreased KAAIs with their assigned footwear, regardless of wedge type

† significant at α = 0.05

**Fig 4:**
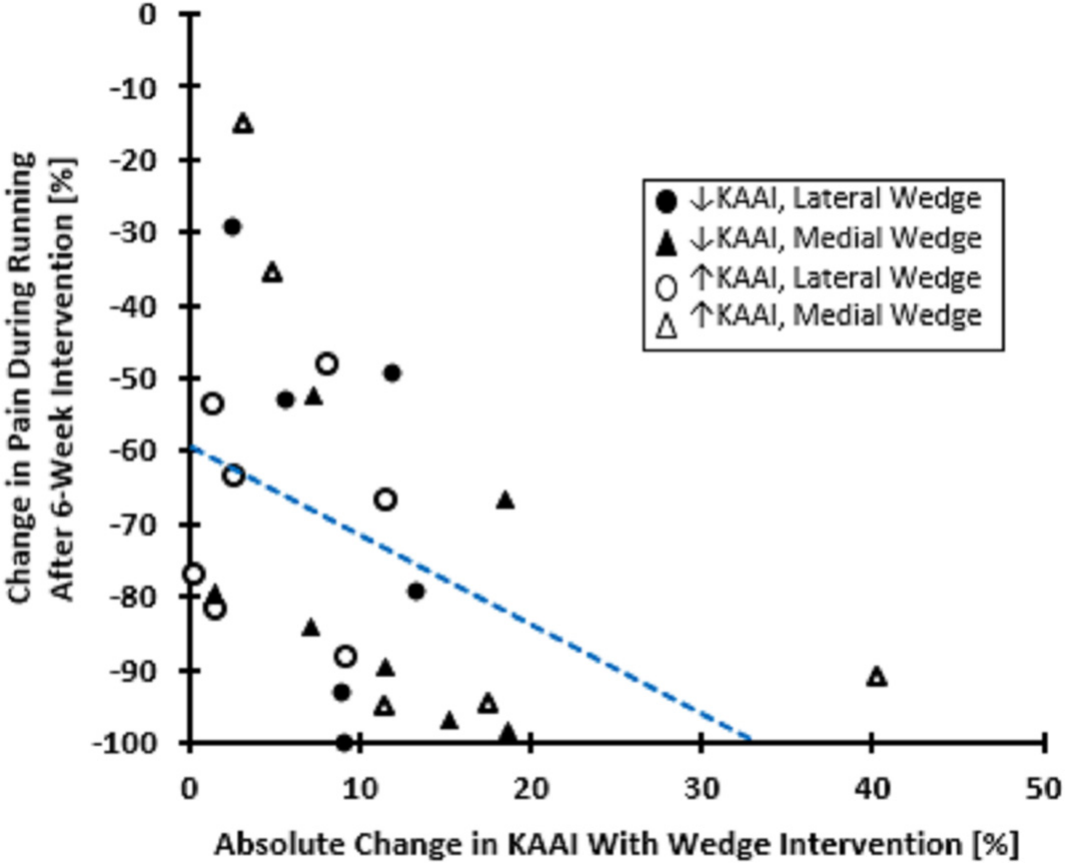
Scatter plot showing relationship between absolute change in knee abduction angular impulse (KAAI) with wedged intervention and pain reduction over 6 weeks. The regression analysis in this article also adjusted for baseline pain; however a 2-dimensional graph is shown here for clarity.

Based on Cook’s Distances (a method of extreme outlier identification that determines data points that incorrectly alter the regression solution), the two participants whose pain increased during running were found to be influential observations and so were removed from the regression model (Participant A: lateral wedge, KAAI increase of 12%, pain increase of 32%; Participant B: medial wedge, KAAI increase of 19%, pain increase of 82%). These two data points also explain the seemingly large difference in pain reduction scores between the decreased KAAI and increased KAAI groups in Table 3. Thus, our regression analyses only included those participants whose pain improved over the 6-week study. Adjusting for baseline pain scores during running, % change in KAAI was not associated with % reduction in pain during running (n = 25, *R* ^*2*^ = 0.019, *p* = 0.915). Since both the increased and decreased KAAI groups experienced pain improvement, a second post-hoc multiple regression analysis was conducted to assess the relationship between % pain reduction and the absolute % change in KAAI instead of using the ± % change in KAAI. Here, a significant relationship between absolute % change in KAAI and % change in pain (or, pain reduction) during running was observed (n = 25, *R* ^2^ = 0.21, *p* = 0.030). Specifically, as absolute % change in KAAI magnitude increased, pain tended to decrease (Fig 4).

### Adherence, Co-interventions & Adverse Events

During the six-week study, 22/27 participants used their assigned intervention 100% of the time and 5/27 used the intervention greater than 70% of the time. Of these five, 4/5 had lateral wedges, and 3/5 experienced increased KAAIs.

Co-intervention use is described in Table 4. There was little difference in co-intervention use between groups, and co-intervention use in general was low, with only 7/27 participants reporting use, most commonly anti-inflammatory medication.

**Table 4:** Participant-reported use of co-interventions for patellofemoral pain during the six-week trial.

	Initial Randomization		Regrouping*		Total
Additional Intervention†	3 mm Lateral Wedge (n = 14)	6 mm Medial Wedge (n = 13)	Decreased KAAI (n = 13)	Increased KAAI (n = 14)	(n = 27)
Active release therapy	0	0	0	0	0
Ice following run	0	2	1	1	2
Knee stabilizing device during run	0	0	0	0	0
Non-specific lower extremity muscle strengthening	1	2	1	2	3
Over-the-counter anti-inflammatory use	2	2	3	1	4
Physiotherapy	0	0	0	0	0
Prescription pain medication	0	0	0	0	0
Yoga	1	1	1	1	2
None	12	8	11	9	20

*subjects were regrouped based on whether they experienced increased or decreased KAAIs with their assigned footwear, regardless of wedge type

†some participants used more than one

Five subjects developed a new injury in the medial wedge group, while only one subject developed a new injury in the lateral wedge group. For the medial wedge group, the injuries were foot pain (n = 1), shin pain (n = 1), blisters (n = 1), ankle pain (n = 2). For the lateral wedge group, the injury was foot pain (n = 1). When grouped by wedge effect, three experienced an injury in the increased KAAI group, and three experienced an injury in the decreased KAAI group.

## Discussion

The purpose of this study was to determine whether altering KAAI magnitude with wedged insoles could reduce pain for runners with PFP, and to directly compare lateral wedge and medial wedge interventions. On average, clinically meaningful pain reductions occurred regardless of insole assigned, and regardless of KAAI effect. Over 6 weeks, Collins et al. [17] reported a reduction in worst pain by just under 20 mm (baseline of 59.4 mm) on a standard visual analogue scale for individuals treated with medial wedges. These participants were asked to use their insoles as much as possible with their daily footwear. In contrast, the present study found worst pain reductions of about 30 points (baseline of 40–50 points) for both the lateral and medial wedge groups over six weeks when participants used the insoles exclusively with their running footwear. Similar to these results, Eng and Pierrynowski reported pain reductions during running of about 66% from week 2 to week 8 (week 0 not reported) with medial wedges [18]; however, this intervention was combined with an exercise program. It should be noted that the construction method of the wedges differed in each study, suggesting construction method may play a role in wedge efficacy.

Differences in pain reduction between the 3 mm lateral wedge and 6 mm medial wedge groups, and between the increased KAAI and decreased KAAI groups were not found in the present study. In addition, no relationship was found between % change in KAAI and % change in pain. Thus, our hypothesis that decreased KAAIs would be associated with greater pain reductions compared to the increased KAAI group was not supported. However, when assessing the relationship between absolute change in KAAI (i.e. regardless of KAAI increase or decrease) and % change in pain, a significant association was found.

Previously, it has been shown that runners with PFP experience higher KAAIs during running than healthy controls [11]. Similar to work on knee osteoarthritis, where increased frontal-plane loads are also implicated in disease progression [31, 32], we expected a decrease in KAAI magnitude to be most beneficial for treatment. However, our results suggested that further increases to KAAIs were beneficial in the short term as well. We have presumed that PFP is a condition of mechanical origin, i.e. where increased patellofemoral loads are implicated in disease development and continuance,[4,11] and that these mechanical changes occur internally, as opposed to external joint angle changes from the wedged insoles [14]. If this is true, then mechanically, PFP treatment would require either decreased joint stress, and/or alteration of the location of peak stresses (i.e. change patellar tracking) [4,38]. Given that both increased and decreased KAAIs seemed beneficial for PFP management in this study, yet long-term increases to the KAAI may have implications for development and progression of medial tibiofemoral osteoarthritis, [31, 32, 39] further investigation into the relationship between KAAI magnitudes/changes and patellofemoral mechanics would be important. This could be done through use of musculoskeletal simulation or *in vivo* animal modeling approaches [40, 41]. It may also be of interest to investigate further the role of the retinaculum in modulating frontal-plane knee joint loads and patellofemoral mechanics.

In the present study, absolute magnitude of KAAI change accounted for 21% of the variability in the observed change (reduction) in pain over six weeks. While this value is low, it is to be expected since KAAI–a tibiofemoral joint variable–was used as a surrogate for patellofemoral joint loading. This was necessary because estimation of patellofemoral loads using motion analysis alone is not possible. In addition, the precise influence that resultant KAAIs have on patellofemoral mechanics is not yet known, as described above. These two points may account for much of the remaining 79% in data variability. At present, KAAIs should not be used clinically as a predictor of PFP outcome until further research is conducted to better understand their relation to patellofemoral mechanics.

The influence of wedge type on KAAI change was not consistent. Specifically, the ratio of subjects who experienced increased KAAIs versus decreased KAAIs was approximately 1:1 for both the lateral and medial wedge groups. In healthy runners, some studies have reported similar inconsistent KAAI effects [19]; however, as our data suggest, the shape of the wedged insole does not seem to be as important as the mechanical effect it produces in terms of pain reduction over six weeks. Other research on insoles have also shown that as knee joint loading variables are altered, it is also possible that altered loading at other joints such as the ankle or hip may occur [14,25]. In the long term, this may have implications for new injury development, and so should be considered in future trials.

### Strengths & Limitations

Strengths of this study included our randomized and blinded design, the high degree of control we implemented that reduced the possibility of measurement bias, our close monitoring of subjects, as well as the collection of biomechanical data in conjunction with pain data, allowing for direct quantification of a treatment mechanism.

Although we achieved high control using standardized wedges with a constant shoe model for all participants, custom-footwear orthotics are often preferred in the clinical setting due to improved comfort, or consideration of other anatomical characteristics [9,17,18,42]. Other studies have shown that custom foot orthotics that incorporate a wedge produce similar joint loading effects as a wedge alone, and so we believe our results are clinically applicable and externally valid [33]. While the controlled shoe model allowed us to attribute all observed mechanical changes to the wedged insole, a limitation with this approach is that we do not know if the shoe alone induced changes to the participant’s baseline KAAI. Consequently, the actual magnitude of KAAI change relative the participant’s baseline in their own shoe may be higher or lower than what is reported in this study, and this may have affected the goodness of fit of our regression model, or possibly even explain why individuals in the increased KAAI group experienced clinical improvement. Additionally, our regression model only considered participants who experienced a reduction in pain, and so the influence of altered KAAIs on worsening symptoms remains unknown.

The primary limitation of this study was a relatively short follow-up period. The six-week period was chosen since this has been shown to be the time of greatest improvement for PFP [17]. Extended follow-up times should be studied to determine the long-term effects of altered KAAIs using medially or laterally wedged footwear. In addition, although our sample size was smaller than other larger long-term trials [17], our sample was sufficient as a trial with the objective being to determine if large treatment differences between groups existed, and to identify the mechanism by which wedged insoles produce clinical benefits.

An additional limitation of this study was that KAAIs were only measured at baseline. Lewinson et al. [43] have recently shown that KAAI magnitudes are maintained during 30 minute runs with medial and lateral wedge insoles, and other authors have shown that KAAI magnitudes are maintained over a period of 6 weeks with wedged insoles [44]. Together, this suggests that under normal circumstances, KAAI magnitudes would not be expected to change over 6 weeks or during a given run. It is not known whether this holds true for clinical populations where KAAI magnitude may interact with pain severity and potentially change if pain changes over time. Consequently, it is possible that one explanation for why we observed improvements in both the increased KAAI and decreased KAAI group is that KAAIs were continually altered during the course of the 6-week study. Since we hypothesized that reduced KAAIs would be superior, we did not anticipate a need to collect follow-up biomechanical data; however, it is recommended that future trials assess biomechanical parameters at baseline and at follow-up to confirm that KAAIs are maintained over the duration of the study.

Finally, the lack of a neutral control group could be seen as a limitation since we were not able to compare both lateral and medial wedges to a neutral group. However, given the success of medial wedges in previous PFP studies [17,18], we assumed the medial wedge to be a supported clinical treatment and instead designed a superiority study for direct comparison of two wedge types rather than withholding participants from receiving treatment, as would be the case in a neutral group.

To further strengthen the external validity of our findings, the mean age, height, mass, body mass index, and sex distributions of the sample in our study were similar to those reported in other PFP trials [17,45].

## Conclusion

Greater absolute % change in KAAI during running was related to greater % reductions in pain over six-weeks for runners with PFP who experienced pain reduction with a wedged insole, regardless of whether lateral or medial wedged insoles were used. This suggests that absolute % change in KAAI is one mechanism by which wedged insoles elicit clinical benefits. This is also the first study to show that laterally wedged insoles have the potential to serve as an alternative therapy to medial wedges for management of PFP; however, they should be studied further in long-term studies before implementation into regular clinical practice.

## Supporting Information

S1 CONSORT Checklist CONSORT Checklist for the study based on the submitted manuscript. (DOC)

S1 Protocol Original protocol approved by the University of Calgary Conjoint Health Research Ethics Board. (DOCX)
