# Supplementary material for: Altering Knee Abduction Angular Impulse Using Wedged Insoles for Treatment of Patellofemoral Pain in Runners: A Six-Week Randomized Controlled Trial
Source: PLoS One. 2015 Jul 31;10(7):e0134461. doi: 10.1371/journal.pone.0134461 (PMC4521888; doi:10.1371/journal.pone.0134461)
Supplement: S1 Protocol — (DOCX) [file pone.0134461.s002.docx]

| **SECTION A – GENERAL INFORMATION** |
| --- |
| **Date received by OMB/CHRO: Ethics ID:** |
| **1. Protocol Title:**  **treatment for pfps using knee abduction Moment reducing footwear**  **2. Local Principal Investigator: Dr. Darren J. Stefanyshyn, PhD**  **(Note: Students, Residents & Fellows cannot be PI)**  **Faculty (rank) and Primary Department: Professor – Kinesiology**  **Phone: (403) 220 - 8637 Fax: (403) 284 -3553 email: darren@kin.ucalgary.ca**  **Co-Investigators: *Ryan T. Lewinson, B.Sc. *Jay T. Worobets, PhD Preston Wiley, MD, MPE**  **(student/fellow with *) MSc student in Biomed. Eng. PDF in Kinesiology Sports Medicine Physician**  **(403) 966-7371 (403) 220-2704 (403) 220-8276**  **rlewinson@kin.ucalgary.ca worobets@kin.ucalgary.ca wiley@ucalgary.ca**  **Research Coordinator/Assistant(s): N/A**  *********PLEASE CHECK THE FOLLOWING IF APPROPRIATE*********  ■ Masters/PhD Project **□** Medical Student Project **□** Undergraduate Project **□** Resident/Fellow Project  **3. Anticipated Start Date: April 2011**  **Anticipated Completion Date: April 2013**  **Anticipated number of subjects (local only): 30**  **4. Check all that apply:** ■ **Clinical □Health Research** ■ **Basic Science □ RRCT**  Covers: Covers: Covers: Prescribed fee paid  Patients + any of: 1. Qualitative method studies 1. Bench science   1. Phase I-IV studies as per 2.Epidemiology 2. Pathology   Health Canada and ICH GCP 3. Proteomics 3. Biomarkers   1. Randomization among 4. Metabolomics   Current standard practice 5. Date-matching   1. Studies of Natural Health 6. Secondary Analysis   Products as per NHPD/HC 7. Technical testing  **5. Location of Research:** (check all that apply)  **□FMC □ACH □Care in the Community □UCMC** ■**Faculty of Kinesiology □TBCC**  **□PLC □SAC □Healthy Communities □HMRC □Faculty of Nursing □Off-site Medical Office**  **□RVH □CBH □Other □CHS** ■**Other**  **6. Proprietary Rights:** (please check one)  ■ **The investigators can alter the protocol according to their judgment and have full rights to information derived from this research and publication of this information**  **□This research is being done for a sponsor who controls the details of the protocol and the rights to the information gathered.**  **Name of sponsor controlling intellectual property: ________________________________________________**  **7. *This serves as application for disclosure of health information to be used in research and I, on behalf of project personnel identified in this document, agree to the following:***  a)to comply with the *Health Information Act* and all regulations under that Act [section 54(1)(a)(i)];  b)to comply with all conditions imposed by the AHB and the University of Calgary relating to the use, protection, disclosure, return or disposal of the health information [section 54(1)(a)(ii)];  c)to comply with all requirements of the AHB and the University of Calgary to provide safeguards, against the identification, direct, or indirect, of an individual who is the subject of the health information [section 54(1)(a)(iii)];  d)to use the health information only for the purpose of conducting the proposed research [section 54(1)(b)];  e)to not publish the health information in a form that could enable the identity of the subject of the health information [section 54(c)];  f)to not attempt to contact the subject of the health information except in accordance with the Act [section 54(d) and 55];  g)to allow the Custodian of health information access as prescribed by the Act [section 54(e)].  _____________________________________________________________________  **Local Principal Investigator’s Signature**  **Section B – Department Approvals**  **Obtain your Department Head’s signature and signatures of all departments/divisions/services whose operations will be affected by your protocol. This is to ensure that prior to commencement of the investigation these individuals have assessed and endorsed the impact of the proposal in their area. This will include reviewing the proposed budget to they can accommodate any additional requirements arising from the protocol.** |

| **Title of proposed research:**  **treatment for pfps using knee abduction Moment – reducing footwear** | | | | |
| --- | --- | --- | --- | --- |
|  | **Department Service** | **Print Name** | **Signature** | **DATE** |
| **□Yes ■No** | **Anesthesia** |  |  |  |
| **□Yes ■No** | **Cardiac Diagnostics** |  |  |  |
| **□Yes ■No** | **QIHI** |  |  |  |
| **□Yes ■No** | **Diagnostic Imaging** |  |  |  |
| **□Yes ■No** | **Health Records** |  |  |  |
| **□Yes ■No** | **HMRC** |  |  |  |
| **□Yes ■No** | **ICU** |  |  |  |
| **□Yes ■No** | **Lab Med & Pathology** |  |  |  |
| **□Yes ■No** | **Neurodiagnostics** |  |  |  |
| **□Yes ■No** | **Nursing Unit** |  |  |  |
| **□Yes ■No** | **Nursing Unit** |  |  |  |
| **□Yes ■No** | **Nursing Unit** |  |  |  |
| **□Yes ■No** | **Nutritional Services** |  |  |  |
| **□Yes ■No** | **Outpatient Services** |  |  |  |
| **□Yes ■No** | **Pharmacy** |  |  |  |
| **□Yes ■No** | **Respiratory Therapy** |  |  |  |
| **□Yes ■No** | **Surgical Services** |  |  |  |
| **□Yes ■No** | **Tumor Group Leader TBCC** |  |  |  |
| **■Yes □No** | **Faculty of Kinesiology** | **David Severson, PhD** |  |  |
| **■Yes □No** | **Sports Medicine Centre** | **J. Preston Wiley, MD, MPE** |  |  |
| **□Yes ■No** | **Other** |  |  |  |

**My signature below acknowledges and accepts the impact (clinical, financial or otherwise) of this research study on my department/division/program/portfolio and I agree with the costs itemized in the study budget**

**David Severson, PhD**

**_____________________________________ _____________________________________ ____________________**

Signature of Department Head/ Print Name Date

Administrative Officer

| **Regulatory Approvals:**   1. **Health Canada** TPD/NHP and Devices (this item applies only to studies involving   drugs/devices or natural health products). Enrolment of subjects cannot start before the  sooner of either (1) receipt of the letter of no objection or (2) written confirmation from the  Principal Investigator to the CHREB that the investigator has confirmed with Health Canada  after a 30 day waiting period that Health Canada has no pending issues with the protocol.  Does the study involve off-label use of therapeutic agents, devices or natural health products  □ Yes **■** No  **If Yes:**  Have you applied for a letter of no objection (LNO) from Health Canada?  **□Yes □No**  **If Yes:** Attach a copy of the LNO or indicate date of submission for HC review; (If NO: you will  need to apply and obtain either a letter of no objection within 30 days or at the expiry of  the 30 day period for objection confirm in writing with Health Canada that they have  completed their review.   1. **Stem Cell Research**   Does the study involve stem cell research?  **□Yes ■No**  **If Yes** have you applied to the SCOC for review and approval?  Please attach letter of approval or indicate date of application.  3. **University of Calgary**  Does the study involve items which may require review and certification by other university  safety and compliance committees?     1. Institutional Biosafety Committee (Biohazards, Health and Safety) 2. Animal Care (research animals) 3. Environmental Health and Safety 4. Radiation Safety Committee   If any of these apply, include a copy of the certificate with your application |
| --- |

| **SECTION C – RESEARCH PROTOCOL SUMMARY**  **The purpose of this section is to outline the scientific structure of your study. Point form is acceptable.**  **All sections must be complete (do not simply refer to sections in your proposal.** |
| --- |
| 1. List a minimum of **3 key words** which describe your study (MeSH recommended). Please include keywords that would be i   interpretable by a non- medical audience.  *For example: (1) cardiology (2) hypertension (3) myocardial infarction*  (1) motion analysis (4) patellofemoral pain  (2) biomechanics (5) footwear treatment intervention  (3) running |
| 2. Does your study involve any of the following (check all that apply)?  ■ Questionnaire □ Interview □ Chart Review □ Database Linkage ■ Bench Science  Has this study received approval from any of the following designated ethics boards in Alberta: CPSA, ACB, U of A, U of L, CREBA?  □ Yes (if Yes, please append letter) ■ No  Has this study received approval from any other REBs  □ Yes (if Yes, please append letter) ■ No |
| 3.Background Rationale:  Running is an activity that nearly 30% of all Canadian adults participate in; however, injuries frequently occur. Most commonly, the runner will suffer from a chronic condition known as patellofemoral pain syndrome (PFPS), which is characterized by general pain in the anterior knee joint. In fact, over one quarter of all running injuries are PFPS in nature and this amounts to over one million Canadians affected annually. Although the exact biological nature of PFPS is not known, it has been shown through a prospective and retrospective biomechanical evaluation of runners that increased knee abduction moments-of-force may be implicated in the development and continuance of PFPS. Therefore, reducing these moments-of-force may be a possible treatment for PFPS patients.  Reducing knee joint loading can be accomplished through footwear intervention. This has been done successfully for healthy subjects and subjects with knee osteoarthritis. For osteoarthritic patients, not only was the knee joint loading reduced, but subjective levels of pain were decreased over time. The effects of these footwear interventions on patients with PFPS are currently not known. |
| 4.Hypothesis/Research Question/Objectives:  Primary Objectives:   1. Determine if a footwear intervention can reduce knee joint loading for runners with PFPS. 2. Determine if this decrease in knee joint loading corresponds to a decrease in subjective levels of pain over time.   Hypotheses:   1. We expect that footwear can decrease knee joint loading for PFPS subjects since this has been done for healthy subjects and patients with knee osteoarthritis. 2. If knee joint loading can be reduced, we expect a decrease in subjective levels of pain over a period of six weeks. |
| 5.Basic Study Design (Briefly describe your study):  Patients will be asked to run across a force platform (which measures ground reaction forces) while high speed cameras record body segment trajectories through space. Together, these data will allow us to noninvasively calculate internal forces at the knee joint. Each patient will be asked to perform trials with a neutral and experimental shoe conditions so that we can determine whether the experimental footwear reduces knee joint loading for PFPS patients. Then, each subject will receive either the neutral or an experimental shoe (this will be randomized and blinded for the patients) to take home for six weeks. The subjects will be asked to continue their training protocol and run on a regular basis with the shoe provided while knee pain levels and running mileage are subjectively monitored. At the end of the study, we will be able to determine if a decrease in knee joint loading is correlated with a decrease in knee pain. |

| 6.Methods:  a) Subject numbers: How many local subjects? 30 How many total subjects? 30  *b) Major Inclusion/Exclusion Criteria:*  All patients must meet the following pre-established inclusion criteria (Lun et al., 2005; Stefanyshyn et al., 2006). These will be assessed by a sports medicine physician prior to beginning testing.  **History:** (1) Nontraumatic unilateral and/or bilateral peripatellar or retropatellar knee pain; (2) Patellofemoral knee pain with and/or after activity; (3) Inactivity patellofemoral pain and/or stiffness, especially with sitting with knees held in flexed posture; (4) No history of knee surgery; **Physical examination:** (1) No or minimal articular or periarticular effusion or bursitis; (2) No significant joint line tenderness; (3) No intra-articular ligamentous instability; (4) Peripatellar tenderness ± mild inferior patellar pole tenderness; (5) No patellar apprehension; **Other:** (1) Between the age of 18 and 45; (2) Run at least 15 km per week regularly; (3) do not have any other medical conditions; (4) are not participating in any other PFPS treatment; (5) Are heel-toe runners  *c) Interventions: (What will be done to the subjects and for how long?)*  **PILOT TEST:** 10-16 healthy subjects will be recruited internally at the Human Performance Lab to pilot test 8 footwear conditions (±3, ±6, ±9 degree orthotic wedge inserted into the neutral shoe [+=medial. - = lateral; these are standard wedge angles, eg, Fisher et al., 2007]; an off-the shelf adidas ForMotion running shoe (allows slight relative movement at the heel of the shoe between the foot and outsole), and an off-the-shelf adidas shoe with ForMotion restricted [this is the neutral condition]). All shoes are displayed on a separate attachment for this application. Subjects will be asked to run at 4 m/s during 5 motion analysis trials with each shoe condition (i.e. 40 trials total). Order will be randomly assigned. In motion analysis trials, reflective markers are attached to anatomical landmarks of the lower extremity. Subjects run over a force platform while infrared cameras record marker trajectories. The 3D positions of the markers are coupled with the ground reaction forces recorded from the force plate, and through a series of inverse dynamics calculations we are able to indirectly determine forces in the knee joint. Specifically, we are interested in the footwear that can most significantly reduce knee abduction moments. The footwear that shows the best optimization between the mechanical benefit just described, and shoe comfort will be chosen as the intervention condition in the following experiment. The test should take between 1.5-2.5 hours per subject. **PFPS STUDY**: 30 patients with PFPS will be asked to perform 10 motion analysis trials in a laboratory setting. The motion analysis procedure will be the exact same as described in the above pilot test. Of these 10 trials, 5 will be with the intervention shoe determined from the above pilot test, and 5 will be with the neutral running shoe described above. Order will be randomly assigned. One or two more trials/conditions may or may not be added if we find multiple shoes may be of benefit from the pilot test results. The total laboratory setting testing should take 1.5-2.5 hours per subject. Half of the patients will then be given the neutral shoe and the other half will be given one (only one) of the experimental shoes to take home and use when running on a regular basis for six weeks. During this time, subjects will be asked to complete weekly questionnaires which will record their current levels of knee pain and daily running mileage. At the end of the six weeks, subjects may be asked to return to the lab for a secondary motion analysis test in their experimental footwear in the event no difference was found in the first lab test, but a decrease in pain was still noted.  *d) Primary Outcome Variables (Briefly describe what will be measured and how/using what measurement tools)*  Knee joint loading will be calculated through inverse dynamics by coupling ground reaction force data from a Kistler force plate (Kistler AG, Winterthur, Switzerland) with kinematic data recorded by infrared motion analysis cameras (Motion Analysis Corp., Santa Rosa, CA). Specifically, we will look at knee abduction angular impulses (integral of the abduction moment-of-force over time) using the KinTrak program (KinTrak 7.0, University of Calgary, Calgary, AB). Subjective levels of pain will be recorded by 10cm visual analogue scale (VAS).  *e) Limitations (please list briefly any limitations you have identified in the study)*  A large amount of research is being done on PFPS patients presently at the University of Calgary, so subject recruitment may be competitive. To overcome this limitation, we will recruit subjects external from the University of Calgary as well as internally. Volunteers for our study will undergo running motion analysis testing, which may be an attractive experience for some runners, thus improving the attractiveness of this study. Secondly, although subjects will be asked to refrain from participating in any other treatment for their PFPS, such as physiotherapy, throughout the study, there is no way to actually control this. Instead, we will remind patients weekly to avoid these other treatment options, and ask patients to provide an honest account of other treatments they used during the six week study period once the study has completed. This way, we will at least be able to factor this into data analysis. |
| --- |
| 7.Statistical Considerations in the study design:   1. *What is your sample size and how do you justify it? (Provide details of sample size calculation)*   Our sample size will be 30 subjects. No pilot test data or data from other researchers’ studies exist in regards to PFPS patient footwear interventions focusing on knee joint loading, thus power analyses cannot be properly performed at this time. However, in most biomechanical studies, a sample size between 10-16 subjects can achieve over 80% power. This is because many biomechanics studies, including the one proposed in this application, employ a paired-samples statistical design, thereby reducing within subject error. Therefore, our planned sample size of 30 should be more than sufficient. Once testing begins, and results from 6-8 subjects are known, full power analyses will be conducted to ensure over 80% power will be reached through 30 subjects. Sample size will be adjusted accordingly.   1. *Data Analysis (Which method of analysis will you use?)*   As mentioned above, the study will use a paired-samples design. Each subject’s motion analysis experimental condition will be compared against their own neutral condition. Thus a paired-samples t-test (a=0.05) will be used here. In the second part of the study, where subjects are asked to run on their own for six weeks, their initial pain score data will be used as a baseline for comparison to their final pain score. Thus, a paired-samples t-test (a=0.05) will be used here as well. |

| 8. Recruitment: Number of Local Subjects: 30 Total number of subjects: 30  a) How will you identify local potential subjects?  Patients with PFPS will be recruited from 1) the Sports Medicine Centre at the University of Calgary upon diagnosis with PFPS; 2) by referral from physician offices in the Calgary area upon diagnosis with PFPS; 3) through poster advertisement in various athletic facilities and sports medicine offices around the Calgary area. All subjects recruited will first undergo assessment by a sports medicine physician at the University of Calgary to ensure the subject has PFPS.  b) Who will recruit the local potential subjects?  Physicians in the Calgary area, physicians in the University of Calgary Sports Medicine Clinic and the investigators of this study by poster advertisement and word of mouth.   1. Where will you recruit potential subjects?   See part (a) above.   1. What method(s) will you use to recruit potential subjects?   Posters will be created and supplied to sports medicine physicians in the Calgary area. We will inform these about our study and request that they pass this information along to their patients with PFPS and provide our contact information to them. We will ask that the physicians mention that our study is a possible treatment for PFPS – an appealing thought considering other treatment forms are limited. We will also inform patients that they would be participating in a personalized running analysis – a potentially exciting experience for avid runners. Lastly, we will offer each subject incentives to participate throughout the study such as free running shoes and running apparel.   1. If you recruitment method involves a letter of invitation, poster, advertisement or electronic announcement, have you included a   copy/copies in this application?  ■ Yes, included □ No □ N/A |
| --- |

| SECTION D – BUDGET SUMMARY To comply with regulatory requirements on the University of Calgary, full budget details are essential. YOUR APPLICATION CANNOT PROCEED WITHOUT BUDGETARY DETAILS. If your study is receiving funding from industry or a granting agency, you must attach a detailed budget. Any funding received from any external source is to be identified. |
| --- |

| 1. Human Resources (List all support personnel to be engaged in the study) | | | | | | |
| --- | --- | --- | --- | --- | --- | --- |
| **Position** | **Salary** | | **Benefits** | **Hrs/Wk** | **Total Hrs.** | **Cost** |
| Graduate student  (Ryan Lewinson)  Sport Medicine Physician  (Dr. Preston Wiley) | $17,500  Volunteer | | N/A | N/A | N/A | $17500  $0  $0  $0  $0 |
|  | | | | | **Subtotal (1)** | **$17500** |
| 2. Service/Procedures (List x-rays ECGs, etc) | | | | | | |
| Service/Procedure | | #Standard Care | | #Research Specific $Cost per item Total | | |
| N/A | | N/A | | N/A $ 0 = $0  X $ = $  X $ = $  X $ = $  X $ = $  X $ = $  X $ = $ | | |
| **Report tests which are usual care but do not include costs for these*  *Tests in your calculations* **Subtotal (2) $0.00** | | | | | | |
| 3. Supplies and Equipment (List all supply and equipment requirements)  Shoes will need to be purchased for up to 30 participants ($100 per shoe x 30 = $3000)  Orthotic wedges MAY need to be purchased if the pre-existing adidas shoe is not used ($20 per wedge x 15 = $300)  Apparel as participation incentives ($20 per t-shirt x 30 = $600)  iPod nano 8GB incentive ($150)  **Subtotal (3) $4050.00** | | | | | | |
| 4.Miscellaneous Items including long term storage and electronic IB preparation  **Subtotal (4) $0.00** | | | | | | |
| 5. Add-on costs: (Please list costs that will not be included in the final total, e.g.. start-up fee)  **COST (Section 1-4): $21550.00** | | | | | | |
| ***Industry Directed Studies Only:*** Overhead (applied to amt rec’d) $N/A | | | | | | |
| REB Review Fee $N/A | | | | | | |
| **TOTAL COST (industry sponsored studies): $N/A** | | | | | | |
| **FUNDING SOURCE(S): Complete sections A and B as applicable** | | | | | | |
| A. **Non-industry Sponsored Funding:**    Canadian Institutes for Health Research – Master’s Award - $17,500 – Received University of Calgary – Faculty of Graduate Studies Scholarship - $3680 – Received | | | | | | |
| **B. Industry Directed Study: N/A** Grant or Contract  Company Name: ____________________________________ Amount per patient $_______________  Contact Person/Phone/Fax/Mail _________________________________________________________  1. Is overhead included in the amount reported above? Yes No Not applicable  2. Will the local investigator or department receive additional funds for participating in this trial? Yes/No | | | | | | |

| **SECTION E – PRIVACY PROTECTION**  **This section must be completed for all research studies. The *Health Information Act* requires an assessment of**  **risks to privacy. Please describe this below. Also describe how you will reduce the risks to privacy (see examples).**  **A copy of *Alberta’s Health Information Act* is available at the public site for Canadian Law: *www.canlii.org*** |  |
| --- | --- |
| ***Project Privacy Management Issues*** |  |
| *Please provide a response/details regarding all of these issues:*   1. List all personal and health information sources and major data elements collected for study use.  - Age - subjective levels of pain - Height - weekly running mileage - Weight - ground reaction force during running - Injury/health history - lower limb kinematics  1. For information source and major data element collected, describe purpose for collection and use of data (please relate   purposes to information listed below).  Personal information (health history, age, weight, height) are used to normalize our subject pool and results by eliminating confounding factors. Also, some are used as inclusion/exclusion criteria for the study. Major data elements such as ground reaction forces, limb kinematics running mileage and subjective levels of pain are needed in order to fully answer our research question: “can footwear reduce knee joint loading and decrease pain for PFPS runners. Collecting kinetic and kinematic data for inverse dynamics calculation is extremely noninvasive and is the best way to quantify knee joint loading. Collecting subjective levels of pain is also very noninvasive and a good estimator of overall trends in pain level over time.   1. List the project personnel who have access to the information listed above.   Darren J. Stefanyshyn Jay T. Worobets  Ryan T. Lewinson J. Preston Wiley   1. Indicate whether any of the information will be disclosed to anyone other than project personnel, or for any purpose(s)   other than the purpose included in this application.  Results will be presented as abstracts at conferences, and published as journal manuscripts. All information relating to individual subject identity will not be disclosed to anyone other than the investigators of this study. Data will be presented as means across subjects rather than displaying results for one individual, so subject identity will remain anonymous. If one subject is focused on, they will be referred to as “Subject 1, Subject 2 etc.” as opposed to using subjects actual names.   1. Does your project involve creating, reviewing or disclosing identifiable subjects’ personal health information (e.g. Health   Records, Charts, images)?  ***NB: This question is not asking whether you will make data anonymous, but whether the information is tied***  ***to identifiable subjects initially so that you would know who the information is about***.  ■ Yes □ No  *If your answer to 5 above, was Yes the Health Information Act of Alberta requires you to obtain informed consent or else to*  *Obtain a waiver of consent from the CHREB. (See CHREB policy and template documents on consent). The Board’s power to*  *grant a waiver is highly circumscribed by law. The Board can grant waivers only on grounds specified under the Health*  *Information Act. If you need a waiver, you must frame your justification incorporating the terms set out in the Health Information*  *Act (currently, section 50). See canlii.org. The only grounds currently for waiver are that it is unreasonable, impractical or not*  *feasible to obtain consent. (Other grounds are not applicable). It is up to the applicants to provide reasons that fall under those*  *headings.*  **EITHER (a) ATTACH PROPOSED CONSENT FORM(S)**  **OR (b) PROVIDE A JUSTIFICATION FOR A REQUEST FOR A WAIVER**  PROPOSED CONSENT FORM IS ATTACHED |  |
| 1. Describe the storage arrangements and final disposition of information collected for research purposes (include destruction   Dates). NB: Under Health Canada requirements clinical trial records must be stored for 25 years after completion of the  study; other studies are covered by common health records practice (12 years) or university research requirements.  The university is developing a uniform policy on storage requirements.  All raw motion analysis data will be initially collected on a communal laboratory computer, but no reference to subject identity will be made. Subject motion analysis data will be stored as “subject 1, subjects 2, etc., or as first initial, last initial eg. John Smith would be saved as JS. Thus, subject identity remains anonymous from the very beginning of the study. The raw data will then be transferred to a password protected folder on a communal laboratory computer, as well as to a private password protected laptop for data analysis. The researchers of this study will retain a subject list which will include subjects full names, age, weight, height and injury status throughout the study. This list will be stored on a private, password protected laptop. Subject pain scores and daily running mileage forms will be sent to the researchers via private email each week. These forms will be saved on a password protected laptop. Once the study is complete, all data will be stored on a private password protected laptop, and in a private password protected folder on the laboratory network for 25 years. All data will be erased in April 2037.   1. Who has access to the information abstracted?   Darren J. Stefanyshyn Jay T. Worobets  Ryan T. Lewinson J. Preston Wiley   1. Who has access to the listing of names and study ID numbers, if there is a study ID number?   Darren J. Stefanyshyn Jay T. Worobets  Ryan T. Lewinson J. Preston Wiley | |

| *Privacy Risks and Controls Assessment* | |
| --- | --- |
| Please provide an assessment of the privacy risks and controls used to mitigate these risks for project, including  The following examples: | |
| ***Risk/Problem***  Unauthorized external or internal access to identifying  Information through:   - Active use - Transmission - Storage - Disposal | ***Mitigation Measures/Solution***   - project personnel screening/agreements - access authorization procedures - designated systems administrator - passwords/screen timeouts - system access audits/disclosure logs - secure mail/transport - firewall/virus protect - encrypted transmission - secure paper-based storage - shredding/wiping |
| Identification through publication or release | - Aggregation levels - Alternate Identifiers |
| Identification through data-matching | Use of non-linkable elements or identifiers |
| Loss of data control outside jurisdiction | Confidentiality and security agreements for out-of-province  Recipients or storage providers |
| Loss of data control through non custodian contractors | Confidentiality and security agreements (e.g. information  Managers, ASPs) |
| Please provide an assessment of privacy risks and controls used to mitigate these risks for the project (you may use  Examples above if applicable):  **RISK:** Unauthorized access to data which may compromise subject anonymity and/or privacy  **SOLUTION:** From the very beginning of the study, subject information will only be stored as “Subject 1, subject 2” etc., or similar so that subject identification is impossible. Motion capture data does not reveal any physical appearance information; motion capture cameras only “see” the reflective markers that are taped to bony landmarks. So viewing this data by an outside source would look like a collection of dots moving freely through space. Moreover, all data will be saved on password protected computers and email accounts. All email correspondence between researchers and subjects (i.e. when subjects send weekly pain reports), will be deleted after they have been saved to the computer. All personal health information other than presence of PFPS will only be revealed to the sports medicine physician, Dr. Preston Wiley – one of the investigators of this study. | |

| **SECTION F – BIOGRAPHICAL SKETCH OF PRINCIPAL INVESTIGATOR** |
| --- |
| □ A recent CV (within 3 years of the date of this application) is on file a Child Health Research Office or the Office of Medical  Bioethics  **OR**  ■ I have included one copy of my CV with this application (current CVs may be submitted electronically to [chreb@ucalgary.ca](mailto:chreb@ucalgary.ca)) |
